# Prediction of Underexpansion Following Transcatheter Aortic Valve Implantation and Efficacy of Balloon Postdilatation

**DOI:** 10.1016/j.shj.2026.100850

**Published:** 2026-04-27

**Authors:** Mariama Akodad, Sri Krishna Sivakumar, Joshua Yoon, Courtney Ream, David A. Wood, Jonathon A. Leipsic, Philipp Blanke, John G. Webb, Lakshmi Prasad Dasi, David Meier, Stephanie L. Sellers

**Affiliations:** a Ramsay Santé, Institut Cardiovasculaire Paris Sud, Hôpital Privé Jacques-Cartier, Massy, France; b Department of Biomedical Engineering, Georgia Institute of Technology, Atlanta, Georgia, USA; c Cardiovascular Translational Laboratory, Centre for Heart Lung Innovation, University of British Columbia, St. Paul’s Hospital & Providence Research, Vancouver, Canada; d Centre for Heart Valve Innovation, St. Paul’s Hospital, Vancouver, Canada; e Centre for Cardiovascular Innovation, University of British Columbia, Vancouver, Canada; f Department of Radiology, University of British Columbia, Vancouver, Canada; g Department of Cardiology, Lausanne University Hospital and University of Lausanne, Lausanne, Switzerland

**Keywords:** Computational modeling, Postdilatation balloon, Transcatheter aortic valve implantation, Transcatheter heart valve dysfunction

## Abstract

**Background:**

Transcatheter heart valve (THV) underexpansion following transcatheter aortic valve implantation (TAVI) is associated with elevated gradients, paravalvular leak, and/or risk of hypoattenuating leaflet thickening. We aimed to simulate TAVI using pre-TAVI computed tomography (CT) images to predict the extent of THV underexpansion and the effect of staged balloon postdilatation on THV expansion.

**Methods:**

Patients who underwent balloon postdilatation as a staged procedure for symptomatic THV dysfunction following native or valve-in-valve TAVI with available pre-TAVI, post-TAVI, and post–balloon aortic valvuloplasty CT between 2016 and 2023 were analyzed. TAVI and postdilatation were simulated computationally in patient-specific models using finite element analysis. Simulations were performed in blinded fashion without information of index procedure. THV expansion was defined as the percentage of THV area achieved by THV in CT or modeling when compared to nominal area defined by THV manufacturer.

**Results:**

Among patients with balloon-expandable THVs following TAVI, the differences in expansion between CT and simulation measurements were −0.88% ± 3.27% (*p* = 0.75), −3.11% ± 4.65% (*p* = 0.26), and −3.27% ± 4.67% (*p* = 0.03) at the inflow, mid, and outflow regions, respectively. Following postdilatation, the differences in expansion between CT and simulation measurements were −0.60% ± 4.7% (*p* = 0.71), −1.64% ± 6.0% (*p* = 0.99), and −1.13% ± 4.2% (*p* = 0.55), respectively. Among patients with self-expandable THVs following TAVI, the percentage difference between CT and simulation measurements were 6.2% ± 4.2% (*p* = 0.99) and 9.3% ± 2.7% (*p* = 0.99) at the inflow and mid regions, respectively. Following postdilatation, the differences in expansion between CT and simulation measurements were 9.0% ± 3.2% and 8.9% ± 3.4%, respectively.

**Conclusions:**

Patient-specific computational modeling can predict THV underexpansion during the index procedure and the effect of postdilatation on THV expansion.

## Highlights

• Patient-specific computational modeling using pre–transcatheter aortic valve implantation computed tomography may accurately predict transcatheter heart valve (THV) underexpansion as well as the effect of late balloon valvuloplasty.
• Computational simulations closely matched post–transcatheter aortic valve implantation and post–balloon aortic valvuloplasty computed tomography measurements of THV expansion across valve regions.
• Predictive modeling identified scenarios in which postimplant balloon valvuloplasty would provide minimal expansion benefit, helping avoid potentially unnecessary risk.
• Predicted and measured THV expansion showed an inverse relationship with post–balloon aortic valvuloplasty mean transvalvular gradients.

## Graphical abstract

Prediction of underexpansion following TAVI and efficacy of postimplant balloon valvuloplasty. Study design (top), methodology of predictive modeling (middle), and results (bottom) showing excellent correlation between CT and predictive computational modeling of THV expansion following balloon aortic valvuloplasty.

**Figure:**
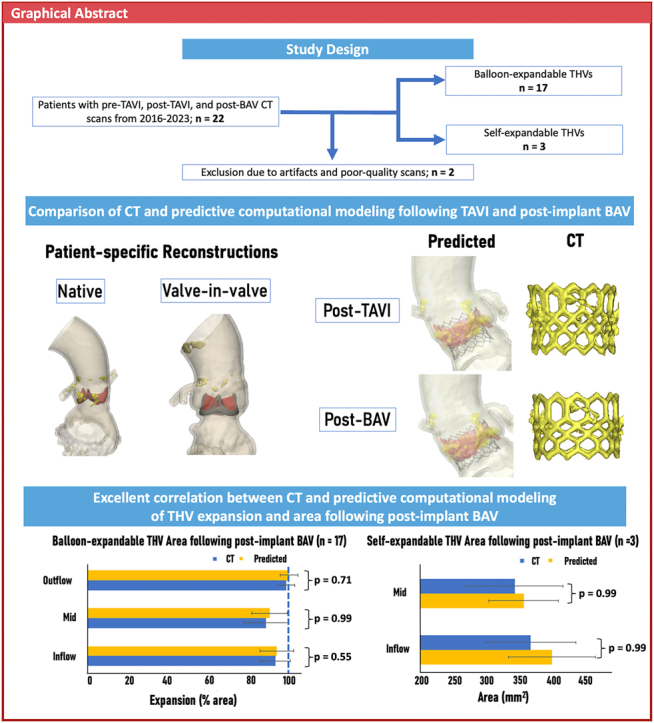

## Introduction

Transcatheter heart valve (THV) underexpansion following transcatheter aortic valve implantation (TAVI) occurs frequently and can lead to elevated residual gradients and/or paravalvular leak (PVL). Underexpansion of THVs has also been shown to increase risk of development of hypoattenuated leaflet thickening (HALT) and can result in THV leaflet redundancy that causes leaflet pinwheeling, potentially increasing leaflet mechanical stress and reducing durability.^1^, ^2^, ^3^

Thus, addressing THV underexpansion during the index TAVI procedure has become an increasingly recognized important clinical consideration. However, reliably identifying THV underexpansion intraprocedurally can be challenging, and real-time evaluation of THV hemodynamics via echocardiography during the procedure may underestimate gradients in sedated patients.^4^^,^^5^ Consequently, intraprocedural decisions regarding additional balloon aortic valvuloplasty (BAV) are generally “in-the-lab” subjective decisions based on operator preferences and experience without the benefit of comprehensive preprocedural planning or detailed consideration of procedural risks.

Computational modeling has emerged as a valuable tool and approach to simulate THV deployment based on individual anatomy to facilitate procedural planning.^6^, ^7^, ^8^ Although recent data on these simulation platforms have focused on predicting THV hemodynamics, procedural complications, and HALT,^7^^,^^9^, ^10^, ^11^ literature on predicting THV underexpansion remains limited in primarily consisting of isolated case reports.^8^^,^^12^^,^^13^ To date, no dedicated studies have simulated or predicted THV underexpansion or evaluated the effects of postimplant BAV. Furthermore, the extent of THV underexpansion and effectiveness of postimplant BAV has not been previously simulated. Therefore, we aimed to simulate TAVI procedures using pre-TAVI computed tomography (CT) imaging to systematically predict THV underexpansion and simulate the impact of postimplant BAV on THV expansion.

## Material and Methods

### Study Population

This single-center retrospective study considered patients who underwent staged balloon postdilatation of an implanted THV following native or valve-in-valve (ViV) TAVI between 2016 and 2023 with available CT scans completed before TAVI, after TAVI, and after BAV. A portion of this population has been previously described in a study examining the safety of late BAV for underexpansion-related THV dysfunction.^3^ The postdilatation balloon was sized at a 1:1 ratio for balloon-expandable THVs and slightly undersized for self-expandable THVs. All postimplant valvuloplasty procedures were performed as stand-alone procedures. This study was approved by the Providence Health Care research ethics board.

### Pre-TAVI, Post-TAVI, and Post-BAV CT Protocols

For pre-TAVI and post-TAVI CT, a dedicated protocol was used; it included intravenous contrast enhancement, electrocardiogram synchronization, and full cardiac cycle acquisition with thin slice (<1 mm) multiphase reconstructions as per the Society of Cardiovascular Computed Tomography recommendations.^14^^,^^15^ The same protocol was used for postimplant valvuloplasty except for intravenous contrast enhancement, which was not systematically performed as previously described.^14^

### Post-TAVI and Post-BAV CT Analysis

THV expansion was assessed on post-TAVI and post-BAV CT at the inflow level and mid-level for all THVs and the outflow level for short-frame THVs (i.e. SAPIEN 3, SAPIEN 3 Ultra) using a standardized segmentation technique (CVI42, circle cardiovascular imaging), yielding area and minimum and maximum diameters. THV expansion for balloon-expandable valves was defined as (THV area on CT/THV area defined by the manufacturer) × 100 at the inflow, mid, and outflow levels and was expressed as a percentage of expansion. The nominal THV areas are 328 mm^2^, 409 mm^2^, 519 mm^2^, and 649 mm^2^ for 20 mm, 23 mm, 26 mm, and 29 mm, respectively.^16^ For the self-expanding valves (i.e., Evolut R, ACURATE neo2), THV area was determined using the mean diameter calculated from the minimum and maximal diameters. All measurements were centralized and performed by the St. Paul’s Hospital core laboratory, whose investigators were blinded to case information.

### Predictive Modeling

#### Computational Modeling Protocol

Pre-TAVI CT images were used to reconstruct the anatomy for computational modeling. The patient-specific models were paired with a model the type and size of the implanted THV, and both the TAVI and post-implant BAV procedures were simulated using finite element methods with Abaqus Explicit 2021 (Dassault Systems, Waltham, Massachusetts). Simulations were performed in blinded fashion without procedural information, such as implantation depth/angle and occurrence of predilatation.

For balloon-expandable valves, THV expansion in each region (inflow, mid, and outflow) was defined as the percentage of THV area achieved in CT or modeling relative to the nominal area defined by the THV manufacturer. THV area, calculated using the mean diameter, was reported for self-expanding valves. All measurements on the computational modeling outputs were performed by the team at Georgia Institute of Technology. All measurements obtained on the CTs were compared to those simulated on the computational models for post-TAVI and post-BAV CTs only after simulations were completed and obtained values were locked in the study database.

#### Echocardiographic Data

The mean gradients measured across the bioprosthetic aortic valve were retrieved from routine transthoracic echocardiograms performed following BAV.

#### Segmentation

**Figure 1:**
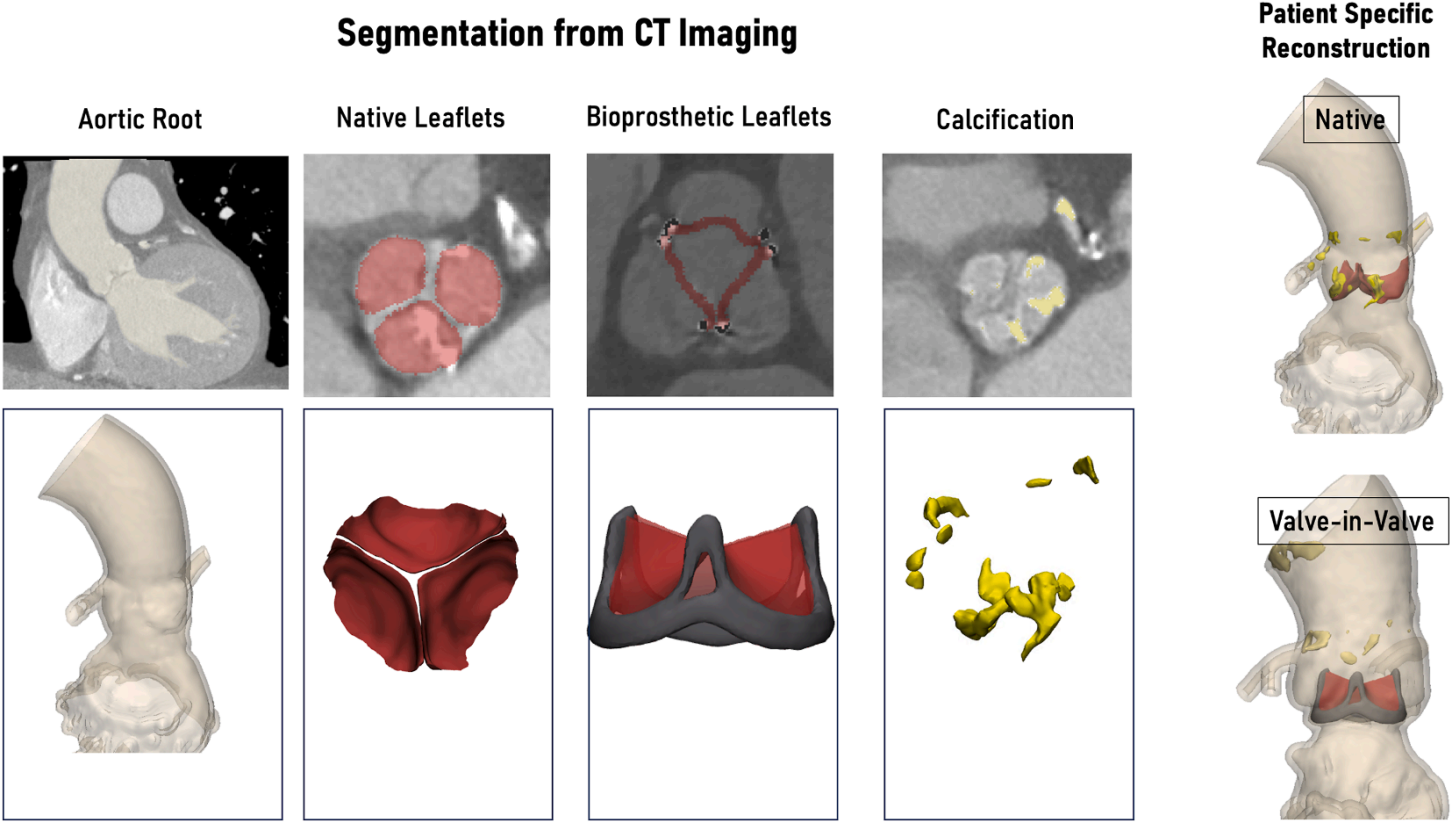
**Patient-specific anatomical reconstruction using pre–transcatheter aortic valve implantation computed tomography imaging in native and bioprosthetic aortic valves.** Abbreviation: CT, computed tomography.

To simulate the THV deployment, patient-specific anatomical models of the aortic root, native or bioprosthetic valve leaflets, bioprosthetic stent, and calcification were reconstructed from pre-TAVI CT imaging using segmentation methods (Figure 1) in Materialise Innovation Suite (Materialise, Leuven, Belgium). The aortic root was segmented by applying a Houndsfield unit threshold of 226 to generate a mask and separating out the blood volume of the ascending aorta, aortic valve, and left ventricle using a multiple slice edit function. Postprocessing of the aortic root was conducted by hollowing the aortic root lumen volume and trimming off the bottom of the ventricle and the top end of the ascending aorta. A reslice plane was defined perpendicular to the aortic annulus to better visualize the leaflet and/or bioprosthetic valve geometry. Native and bioprosthetic aortic valve leaflets were then segmented by removing the left ventricle from the aortic root mask and capturing the lumen volume above the leaflets. A Boolean subtraction method was applied to the leaflet mask to isolate the 3 native or bioprosthetic valve leaflets. Patients with thickened native or bioprosthetic leaflets were segmented directly from the CT images. A higher Houndsfield unit threshold, variable based on level of contrast, was applied to generate a mask containing the calcification and/or bioprosthetic valve stent. They were separated from the rest of the mask using a region grow function that allows for selective extraction of isolated volumes.

#### Simulation

The THV stent geometries were created in SolidWorks 2021 (Dassault Systems) using measurements extracted from micro-CT images. The balloon geometries were created in Abaqus Explicit 2021 (Dassault Systems) using *in vitro* measurements. Abaqus Explicit 2021 is a finite element analysis software capable of simulating complex interactions and dynamic events, used here to computationally simulate THV deployment mechanics. To virtually deploy the balloon-expandable SAPIEN THV, the balloon and SAPIEN stent geometries were computationally crimped to a diameter of 6 mm, then positioned at the simulated center of the aortic valve annulus using nominal deployment parameters. The fluid-cavity method in Abaqus Explicit 2021 was used to virtually inflate the balloon to deploy the stent, followed by balloon deflation to allow the stent to virtually recoil and settle into its projected final position. For self-expandable THVs, the simulated stent was similarly crimped and positioned and then virtually deployed by computationally removing the sheath in a controlled vertical fashion, allowing for the self-expandable valve to expand and interact with the simulated native aortic root, bioprosthetic or native leaflets, and calcification.

To simulate the standalone postdilatation of the THV, the simulated post-TAVI anatomical models and stent were imported into Abaqus, and a balloon was positioned at the center of the THV stent. Postdilatation of the THV was simulated using balloon models created with diameters corresponding to the size of the balloon used for postimplant BAV and material properties modeling the noncompliant balloon behavior. Prosthesis deformation index was calculated using the diameter measurements at the ends of the THV frame (inflow and outflow) and the prosthesis mid region as previously described.^16^ Additional details regarding predictive modeling are provided in the supplemental methods.

### Statistical Analysis

Continuous variables were tested for normal distribution and reported either as mean with SD or as median with interquartile range (IQR). Categorical variables were reported as frequencies and proportions. Comparisons between pre-BAV and post-BAV CT parameters were performed using McNemar test for nominal variables, Wilcoxon matched-pairs signed-rank test for ordinal variables, and analysis of variance or the Kruskal–Wallis test for continuous variables. Effective postimplant BAV was defined as any statistically significant increase in measured expansion or area compared to post-TAVI measurements.

Comparisons of the mean diameter and THV expansion between the post-TAVI, post-BAV CT measurements and the post-TAVI, post-BAV computational models were performed using the Kruskal–Wallis test, with *p* < 0.05 considered statistically significant. Simple linear regression and Pearson correlation coefficients were calculated between predicted/actual expansion on CT and the mean gradient determined on echocardiogram after BAV, with *p* < 0.05 considered statistically significant. Influential observations were identified using Cook’s distance derived from the linear regression model. Observations with Cook’s distance greater than 4/*n* (where *n* represents the sample size) were considered influential and evaluated in sensitivity analyses.

Prosthesis deformation index has been associated with HALT; this was calculated as previously described (1). For balloon-expandable THVs, Bland–Altman plots of the difference in THV expansion were generated to assess the agreement between the simulated and post-TAVI/post-BAV stent geometries, with red lines indicating the 95% limits of agreement (LOA), calculated as mean difference ± 1.96 × SD. All reported *p* values were two-sided, and statistical significance was set at *p* < 0.05. All analyses were performed using STATA 16.1 (College Station, Texas).

## Results

### Baseline and Procedural Characteristics

There was a total of 22 patients who underwent postimplant BAV with available pre-TAVI, post-TAVI, and post-BAV CTs. Of these, 2 patients had pre-TAVI CTs that were unavailable for computational modeling due to artefacts and poor-quality images. Baseline characteristics are presented in Table 1. The median age was 74.7 (71.4–82.5) years, and native TAVI and ViV TAVI were performed in 11 patients (55%) and 9 patients (45%), respectively. The median delay between TAVI and post-TAVI CT (n = 20) was 2 months [IQR: 1, 4.5]. The median delay between BAV and post-BAV CT (n = 20) was 14 days [IQR: 0, 54]. HALT was not reported in 5 pre-BAV CT scans. Furthermore, 3 patients received a self-expanding valve, whereas 17 patients received a balloon-expandable valve. Further characteristics of the initial THV implantation are shown in Table 2. Cases were implanted at nominal volume with the exception of 2. BAV was performed 3.5 (2–7) months following the index TAVI procedure. Indications for the BAV procedure are provided in Supplemental Table 1, with the most common indications being for elevated mean gradients (60%) and PVL (35%). The majority of patients required only 1 balloon inflation (60%), with >2 inflations required in 20% of cases. One patient had a bicuspid aortic valve. Notably, bioprosthetic valve fracture was never attempted in any of the ViV cases. Baseline clinical characteristics are noted in Supplemental Table 2. The average percentage oversized was 7.3% for native TAVIs (n = 9) and was also stratified by percentage oversized: 0%-5% (n = 4), 5%-10% (n = 2), >10% (n = 3).

**Table 1:** Initial THV implantation procedure characteristics

	Total population n = 20
Age	74.7 [71.4–82.5]
Male	13 (65%)
Valve-in-valve procedure	9 (45%)
Balloon-expandable THV	8 (40%)
Self-expandable THV	1 (5%)
Balloon-expandable THV	17 (85%)
Sapien 3	8 (40%)
Sapien 3 Ultra	9 (45%)
Self-expandable THV	3 (15%)
Evolut R	2 (10%)
ACURATE neo2	1 (5%)
Balloon-expandable deployment balloon	
Nominal volume	15 (75%)
Underfilling∗	2 (10%)
Postdilatation†	2 (10%)

Abbreviations: IQR, interquartile ranger; THV, transcatheter heart valve.

∗ (−2cc) for calcification in the right coronary cusp.

† Only in self-expandable valve for native aortic stenosis. Values are median ± IQR for age and n (%).

**Table 2:** Pre-existing bioprosthetic valve type and size (if applicable), THV type and size, and postimplant BAV balloon type and size used for each case

Case number	THV device	Size	Surgical/THV type	Size	Balloon used for BAV
1	Evolut R	26 mm	Magna	23 mm	TRUE 23 mm
2	SAPIEN 3	23 mm	Magna	23 mm	TRUE 23 mm
3	SAPIEN 3	26 mm	Magna Ease	25 mm	TRUE 26 mm
4	SAPIEN 3	26 mm	Perimount	25 mm	TRUE 26 mm
5	SAPIEN 3	23 mm	Trifecta	23 mm	TRUE 22 mm
6	SAPIEN 3	26 mm	N/A - Not ViV	N/A - Not ViV	TRUE 26 mm
7	SAPIEN 3	23 mm	N/A - Not ViV	N/A - Not ViV	TRUE 23 mm
8	SAPIEN 3	26 mm	N/A - Not ViV	N/A - Not ViV	TRUE 25 mm
9	S3 Ultra	26 mm	N/A - Not ViV	N/A - Not ViV	TRUE 26 mm
10	S3 Ultra	26 mm	Magna Ease	25 mm	TRUE 26 mm
11	S3 Ultra	26 mm	Perimount	25 mm	TRUE 26 mm
12	ACURATE neo2	27 mm	N/A - Not ViV	N/A - Not ViV	ZYMED 25 mm
13	Evolut R	23 mm	N/A - Not ViV	N/A - Not ViV	TRUE 22 mm
14	S3 Ultra	23 mm	N/A - Not ViV	N/A - Not ViV	TRUE 23 mm
15	S3 Ultra	26 mm	N/A - Not ViV	N/A - Not ViV	TRUE 24 mm
16	S3 Ultra	23 mm	Trifecta	23 mm	TRUE 22 mm
17	SAPIEN 3	29 mm	N/A - Not ViV	N/A - Not ViV	TRUE 28 mm
18	S3 Ultra	26 mm	N/A - Not ViV	N/A - Not ViV	TRUE 25 mm
19	S3 Ultra	23 mm	N/A - Not ViV	N/A - Not ViV	TRUE 22 mm
20	S3 Ultra	23 mm	Perimount	21 mm	TRUE 22 mm

Abbreviations: BAV, balloon aortic valvuloplasty; N/A, not applicable; S3, SAPIEN 3; THV, transcatheter heart valve; ViV: valve-in-valve.

### THV Expansion on CT

CT analysis of THV expansion in the population for native TAVI and ViV procedures are presented in Table 3.

**Table 3:** Post-TAVI and post-BAV CT analysis for paired THVs

	Post-TAVI CT	Post-BAV CT	Difference pre/post-BAV	*p* Value
All THVs (n = 20)				
Mean diameter, mm				
Inflow	22.4 (21.1–24.1)	23.3 (22.1–25.1)	1.00 (0.60–1.39)	<0.001
Mid	20.6 (18.2–22.6)	23.1 (20.6–25.3)	2.64 (1.19–3.61)	<0.001
Balloon-expandable THVs (n = 17)				
THV expansion, %				
Inflow	87.3 (79.8–91.6)	92.9 (86.6–99.9)	7.2 (4.9–9.7)	<0.001
Mid	66.8 (61.7–79.6)	93.6 (78.6–96.6)	21.7 (10.6–25.3)	<0.001
Outflow	93.9 (90.0–96.5)	99.1 (95.8–102.7)	6.5 (0.9–10.4)	<0.001
Mean diameter, mm				
Inflow	22.8 (21.6–24.6)	23.3 (22.6–25.3)	1.00 (0.60–1.4)	<0.001
Mid	20.7 (18.6–22.3)	23.5 (21.3–25.4)	2.72 (1.6–3.8)	<0.001
Outflow	24.1 (22.6–25.0)	25.2 (23.1–26.3)	0.92 (0.1–1.4)	<0.001
Valve-in-valve (balloon-expandable in surgical heart valves) (n = 8)				
THV expansion, %				
Inflow	81.4 (77.1–86.6)	92.1 (82.5–98.6)	8.2 (5.4–11.2)	0.008
Mid	61.7 (60.9–64.6)	80.6 (73.1–95.6)	22.6 (8.4–32.4)	0.008
Outflow	95.9 (94.0–97.6)	102.7 (97.7–103.5)	6.4 (0.3–9.2)	0.039
Mean diameter, mm				
Inflow	21.6 (21.0–23.0)	23.1 (22.1–23.5)	1.0 (0.7–1.6)	0.008
Mid	19.4 (17.9–20.8)	22.3 (20.4–24.6)	3.1 (1.3–4.7)	0.008
Outflow	23.8 (22.6–25.4)	24.9 (22.7–26.5)	0.8 (0.0–1.2)	0.039
Evolut R (n = 2)				
Mean diameter, mm				
Inflow	18.7 (17.6–20.7)	20.4 (18.9–22.0)	1.7 (1.3–2.2)	-
Mid	17.4 (16.7–18.1)	19.3 (18.4–20.30)	2.0 (0.3–3.7)	-
ACURATE neo2 (n = 1)				
Mean diameter, mm				
Inflow	23.0	23.6	0.6	-
Mid	22.8	23.6	0.8	-

THV expansion (% area) and mean THV diameter assessed by post-TAVI and post-BAV CT in all THVs, including balloon-expandable THVs, valve-in-valve configurations, and self-expandable THVs. Outflow was not analyzed for self-expandable THVs.

Abbreviations: BAV, balloon aortic valvuloplasty; CT, computed tomography; n, number of patients; TAVI, transcatheter aortic valve implantation; THV, transcatheter heart valve.

### Balloon-Expandable Valves

THVs were underexpanded on CT, with expansion significantly improving in all measured levels in balloon-expandable THVs following postimplant BAV. Before BAV, balloon-expandable THVs showed the greatest degree of underexpansion at the mid-level, with a median expansion of only 66.8% of the nominal valve area. Following postimplant BAV, THV expansion improved significantly at the inflow (+7.2%; *p* < 0.001), mid (+21.7%; *p* < 0.001), and outflow (+6.5%; *p* < 0.001). Correspondingly, the mean THV diameter significantly increased at all 3 levels (*p* < 0.001). Prosthesis deformation index of balloon-expandable THVs improved from 1.17 ± 0.06 to 1.03 ± 0.07 (*p* < 0.001) after BAV in native TAVI.

### Self-Expanding Valves

In total, 3 cases with self-expanding valves were included in the CT analysis. Among the 2 cases with Evolut R, the mean diameter increased in the inflow and mid regions (1.7 and 2.0 mm, respectively) but did not reach statistical significance. Similarly, the case with ACURATE neo2 showed an increased mean diameter in the inflow and mid regions (0.6 and 0.8 mm, respectively).

### ViV Procedures

For patients undergoing aortic ViV procedures (n = 9), postimplant BAV also led to significant improvements, particularly at the inflow (+8.71%; *p* < 0.001) and mid-levels (+17.3%; *p* < 0.001). Prosthesis deformation index of balloon-expandable THVs improved from 1.43 ± 0.10 to 1.18 ± 0.18 (*p* = 0.002) after postimplant BAV in ViV TAVI.

### Computational Modeling of THV Expansion

Pre-TAVI CT imaging was available and of sufficient quality in 20 of the 22 patients and was used to reconstruct the 3D anatomical geometry for computational modeling. Of the 20 cases modeled computationally, 11 received TAVI in the stenotic native aortic valve (55%) and nine received TAVI in a failed surgical valve (45%).

### Balloon-Expandable THV Simulations

**Figure 2:**
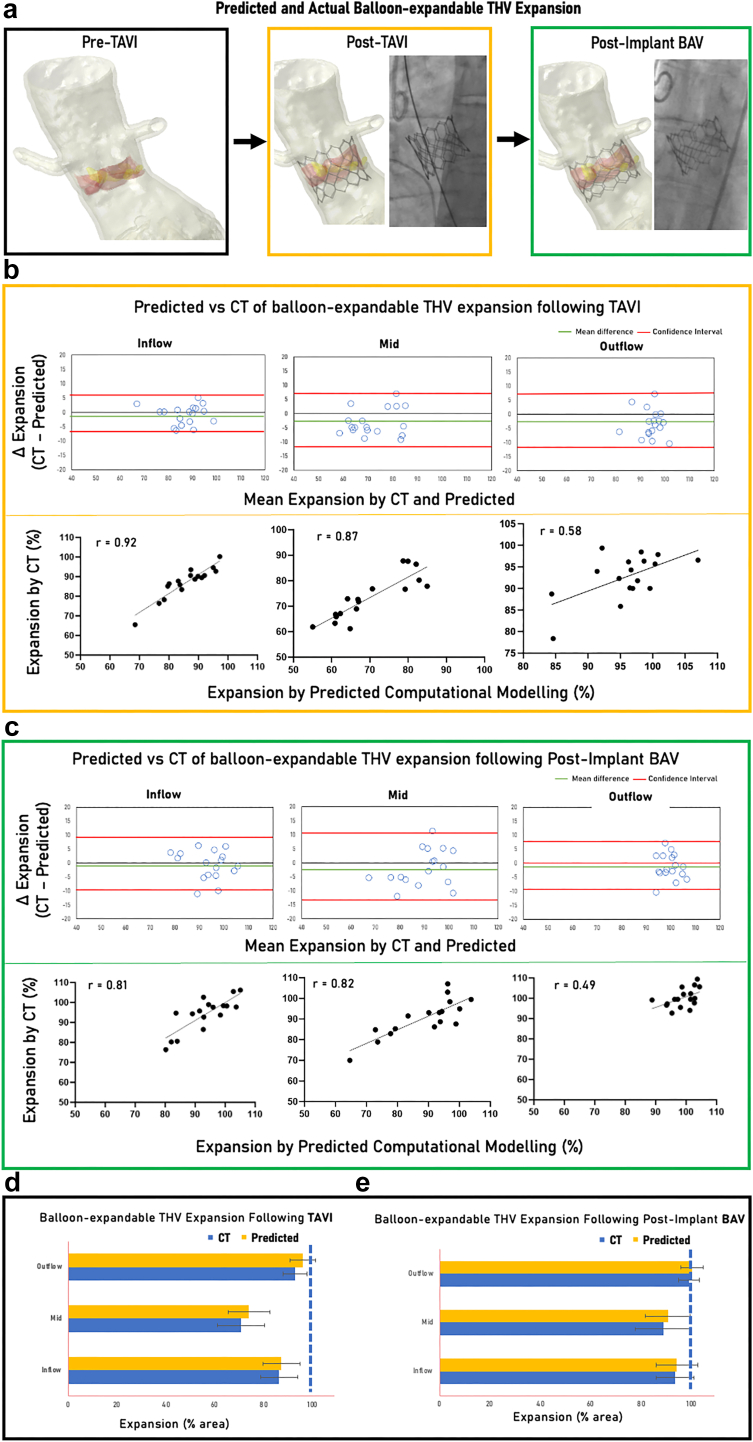
(**a**) Predicted and actual THV expansion of balloon-expandable THVs. Predicted compared to CT analysis for balloon-expandable THVs (**b**) post-TAVI and (**c**) following postimplant valvuloplasty at the inflow, mid, and outflow regions. Expansion determined by CT and computational modeling are similar following (**d**) TAVI and (**e**) postimplant BAV. Abbreviations: BAV, balloon aortic valvuloplasty; CT, computed tomography; TAVI, transcatheter aortic valve implantation; THV, transcatheter heart valve.

The predicted mean diameter and THV expansion for balloon-expandable valves are presented in Table 4 for both the index TAVI and post-BAV computational modeling (Figure 2). Predicted THV expansion from computational modeling in balloon-expandable THVs showed good agreement with the CT analysis with a mean difference of −0.88% ± 3.27% (95% LOA: −7.29, 5.53; r = 0.90; *p* < 0.001), −3.11% ± 4.64% (95% LOA: −12.20, 5.98; r = 0.86; *p* < 0.001), and −3.27% ± 4.67% (95% LOA: −12.42, 5.88; r = 0.59; *p* = 0.014) at the inflow, mid, and outflow regions post-TAVI, respectively (Table 5). For the post-TAVI outflow region, removal of an outlier identified using Cook’s distance attenuated the association (r = 0.36, *p* = 0.17). Following late BAV, predicted THV expansion from computational modeling in balloon-expandable THVs in the inflow, mid, and outflow regions showed good agreement with the CT analysis with a mean difference of −0.60% ± 4.71% (95% LOA: −10.42, 9.08; r = 0.76; *p* < 0.001), −1.64% ± 5.99% (95% LOA: −14.21, 10.53; r = 0.82; *p* < 0.001), and −1.13% ± 4.16% (95% LOA: −9.85, 7.32; r = 0.487; *p* = 0.047), respectively (Table 5). For the post-BAV outflow region, removal of 2 outliers identified using Cook’s distance slightly increased the association but was not statistically significant (r = 0.50, *p* = 0.056). The correlation between THV expansion measured by CT and predicted by computational modeling segregated by native and ViV TAVI is shown in Supplemental Figure 1.

**Table 4:** TAVI and postimplant BAV computational modeling for THVs

	TAVI procedure	Post-BAV	Difference TAVI/BAV	*p* Value
All THVs (n = 20)				
Mean diameter, mm				
Inflow	23.0 (17.3–29.0)	23.9 (19.9–29.8)	0.9 (−0.8 to 2.60)	0.18
Mid	21.2 (18.0–25.6)	23.4 (19.2–27.2)	2.18 (0.4–5.5)	<0.01
Balloon-expandable THVs (n = 17)				
THV expansion, %				
Inflow	87.2 (65.5–100.3)	93.5 (76.4–106.2)	7.1 (−4.5 to 20.0)	<0.01
Mid	73.9 (61.2–87.8)	88.7 (70.0–107.1)	17.2 (2.9–41.8)	<0.001
Outflow	96.0 (84.4–106.9)	98.9 (92.7–109.4)	4.2 (−3.5 to 12.8)	0.03
Mean diameter, mm				
Inflow	23.5 (21.0–29.0)	24.2 (21.4–29.8)	0.68 (−0.8 to 2.3)	0.23
Mid	21.4 (18.0–25.6)	23.7 (19.2–27.2)	2.36 (0.4–5.6)	<0.01
Outflow	24.5 (21.4–27.8)	24.9 (22.2–28.3)	0.38 (−0.4 to 1.5)	0.32
Evolut R (n = 2)				
Mean diameter, mm				
Inflow	19.7 (17.3–22.1)	21.4 (19.9–23.0)	2.3 (2.0–2.6)	-
Mid	19.6 (19–20.2)	20.8 (19.7–21.8)	1.3 (1.0–1.6)	-
ACURATE neo2 (n = 1)				
Mean diameter, mm				
Inflow	22.8	24.3	1.5	-
Mid	21.4	23.0	1.6	-

Abbreviations: BAV, balloon aortic valvuloplasty; n, number of patients; TAVI, transcatheter aortic valve implantation; THV, transcatheter heart valve.

**Table 5:** CT analysis comparison with computational modeling of prestandalone and poststandalone BAV for balloon-expandable THVs

THV level	Post-TAVI CTA	Simulated THV	Difference (CT-simulated)	*p* Value
Prestandalone BAV SAPIEN THV expansion (%) (n = 18)				
Inflow	86.32 ± 7.51	87.20 ± 7.58	−0.88 ± 3.27	0.75
Mid	70.80 ± 9.65	73.91 ± 8.56	−3.11 ± 4.64	0.26
Outflow	92.77 ± 5.00	96.04 ± 5.26	−3.27 ± 4.67	0.03
Poststandalone BAV SAPIEN THV Expansion (%) (n = 17)				
Inflow	93.48 ± 7.51	94.15 ± 8.35	−0.60 ± 4.71	0.71
Mid	88.69 ± 10.93	90.53 ± 8.81	−1.64 ± 5.99	0.99
Outflow	98.92 ± 4.17	100.19 ± 4.46	−1.13 ± 4.16	0.55

Abbreviations: BAV, balloon aortic valvuloplasty; CT, computed tomography; CTA, computed tomography angiography; n, number of patients; TAVI, transcatheter aortic valve implantation; THV, transcatheter heart valve.

Similar to what was observed on clinical CTs, THVs had significant underexpansion predicted in the mid and inflow regions before BAV and THV expansion showing significant improvement after BAV in all 3 levels. Improvement in THV expansion from postimplant BAV was found to be greater in ViV TAVI compared to native valves, with the biggest improvement at the mid region of 21.47% ± 11.15% on average. Prosthesis deformation index of balloon-expandable THVs improved from 1.16 ± 0.06 to 1.05 ± 0.07 (*p* < 0.01) after postimplant BAV in native TAVI and from 1.38 ± 0.11 to 1.11 ± 0.12 (*p* < 0.01) in ViV TAVI.

Interestingly, simulations predicted the patients in whom postimplant BAV would result in the most and least improvement in expansion as illustrated by the four following scenarios.

1. Case 2 involved a ViV procedure using a 23-mm Sapien 3 THV in a 23-mm Edwards Perimount Magna valve, expanded using a TRUE 23-mm balloon. CT imaging demonstrated a substantial 31.41% increase in the mid region (from 64.8% to 96.2%), closely mirrored by the simulation prediction of a 41.81% increase (from 61.2% to 103.0%).
2. Case 3, another ViV scenario, involved a 26-mm Sapien 3 THV implanted within a 25-mm Magna Ease valve, dilated with a 26-mm TRUE balloon. CT showed an increase in expansion by 32.70% at the mid region (from 61.1% to 93.8%), whereas simulation projected a similar but slightly lower increase of 22.81% (from 65.9% to 88.7%).
3. Case 11, which involved a ViV procedure with a 26-mm Sapien 3 Ultra within a 25-mm Perimount using a 26-mm TRUE balloon, showed only a 7.18% increase via CT (from 66.4% to 73.6%), with simulation closely predicting a 9.91% increase (from 69.0% to 78.9%).
4. Case 16 involved a ViV procedure with a 23-mm Sapien 3 Ultra within a 23-mm Trifecta valve, expanded by a 22-mm TRUE balloon. This resulted in minimal area expansion, with a mere 2.38% increase on CT (from 62.2% to 64.6%), closely predicted by simulation (2.9% increase from 67.1% to 70.0%).

To clarify whether accuracy prediction would differ according to the magnitude of change seen on CT, the balloon-expandable THVs were divided into 2 equal groups based on amount of expansion following postimplant BAV. The median difference between CT and simulated expansion was 4.26% [IQR: 2.21–8.62] in the bottom half compared to 6.09% [IQR: 2.46–10.27] in the top half, demonstrating no significant difference in accuracy between the 2 groups (*p* = 0.423; Supplemental Figure 2). Both the predicted and actual expansion of the THVs at the mid region showed a statistically significant negative correlation with the mean gradient measured on echocardiography after BAV (*p* < 0.05; Supplemental Figure 3). Calcium volume was measured from patient-specific segmentations (Supplemental Table 3), with the computational model showing no significant difference in predictive accuracy of the measured expansion at the different THV levels (inflow, mid, and outflow) depending on the calcium load (Supplemental Figure 4).

### Self-Expandable THV Simulations

**Figure 3:**
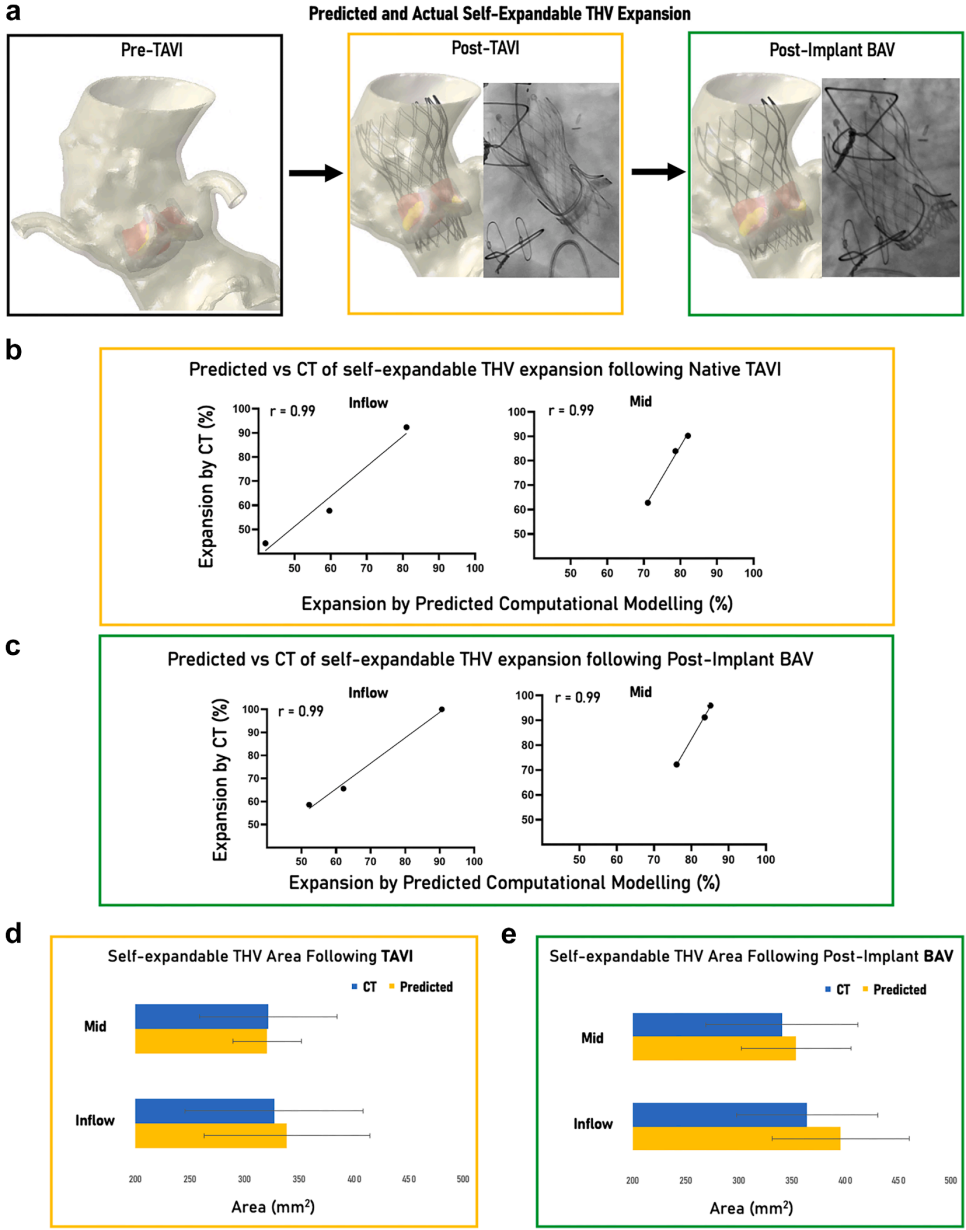
(**a**) Predicted and actual THV expansion of self-expandable THVs. (**b-e**) A comparison of simulated and computed tomography analysis of THV expansion post TAVI and post-balloon valvuloplasty for self-expandable THVs (n = 3). Abbreviations: BAV, balloon aortic valvuloplasty; CT, computed tomography; TAVI, transcatheter aortic valve implantation; THV, transcatheter heart valve.

**Figure 4:**
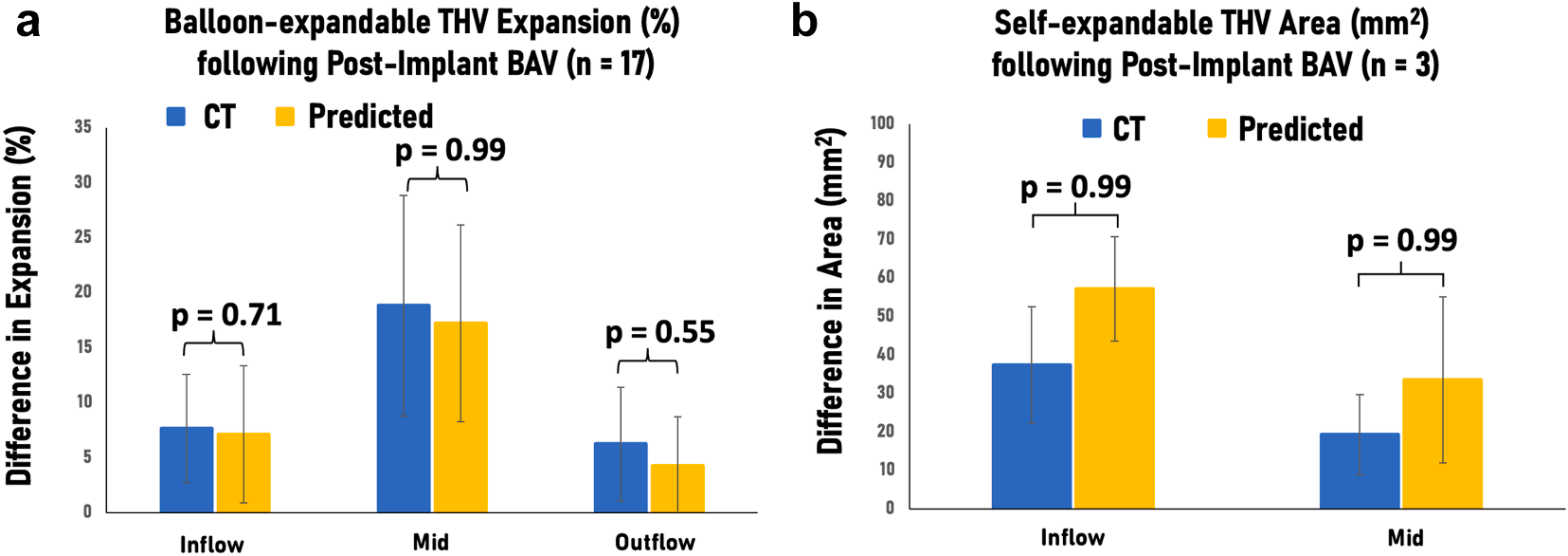
Nonsignificant difference in expansion and area predicted by computational modeling and CT analysis in THV following (**a**) postimplant BAV for balloon- and (**b**) self-expandable THVs. Abbreviations: BAV, balloon aortic valvuloplasty; CT, computed tomography; THV, transcatheter heart valve.

Comparison of the self-expandable THV area between computational modeling and the corresponding CT showed a mean difference of 6.23% measured at the inflow and 9.36% at the mid (Table 6) for the TAVI and 9.07% at the inflow and 8.95% at the mid after BAV (Figure 3). The overall improvement in expansion of balloon-expandable THVs and improvement in THV area of self-expandable THVs were similar to those simulated computationally (Figure 4).

**Table 6:** CT analysis comparison with computational modeling prestandalone and poststandalone BAV for self-expandable THVs

Case	THV	THV level	Post-TAVI CTA	Simulated THV	% difference (CT-simulated)
Prestandalone post-BAV self-expandable THV area (mm^2^) (n = 3)					
2	Evolut R 26 mm	Inflow	222.50	233.5	4.94
		Mid	298.82	318.3	6.52
14	ACURATE neo L	Inflow	421.50	409.19	2.85
		Mid	407.00	359.5	11.8
15	Evolut R 23 mm	Inflow	336.40	372.94	10.9
		Mid	257.7	282.88	9.77
Poststandalone post-BAV self-expandable THV area (mm^2^) (n = 3)					
2	Evolut R 26 mm	Inflow	277.0	308.7	11.4
		Mid	323.4	363.2	12.3
14	ACURATE neo L	Inflow	439.3	463.11	5.42
		Mid	435.5	411.8	5.44
15	Evolut R 23 mm	Inflow	376.4	415.4	10.4
		Mid	262.4	286.2	9.1

Abbreviations: BAV, balloon aortic valvuloplasty; CT, computed tomography; CTA, computed tomography angiography; n, number of patients; TAVI, transcatheter aortic valve implantation; THV, transcatheter heart valve.

## Discussion

For the first time, this study used a specific patient cohort to evaluate the ability of computational modeling to predict THV expansion following TAVI as well as the efficacy of postimplant BAV. The main findings are the following: (1) a statistically significant and reproducible correlation was observed between actual and predicted post-TAVI THV dimensions; (2) the models accurately predicted post-BAV THV dimensions; and (3) the model allowed identification of cases in which postimplant BAV would have limited effect, with no significant differences in simulation accuracy between those with the most and least benefit (Graphical Abstract). This study thus provides initial evidence supporting the use of computational modeling to predict post-TAVI and post–implant-BAV valve geometry and may help guide clinical decision-making to optimize THV expansion.

Achieving optimal expansion at time of index procedure is increasingly recognized as an important factor in long-term valve performance.^7^^,^^10^^,^^11^^,^^17^^,^^18^ There is emerging evidence that many, if not all, balloon-expandable THVs may experience some degree of underexpansion after the first inflation despite nominal deployment.^19^ THV underexpansion is linked to accelerated degeneration, higher gradients, and restricted leaflet mobility, which may not be immediately apparent during on-table echocardiography. Postimplant BAV of underexpanded THVs may improve leaflet durability and reduce the risk of leaflet thrombosis or HALT by restoring a more optimal frame geometry and promoting optimal leaflet coaptation.^3^ By identifying regions of suboptimal expansion through computational modeling, operators may proactively mitigate these risks. Furthermore, HALT has been linked to areas of low flow and suboptimal leaflet kinematics, which may be exacerbated by underexpansion; optimizing THV expansion could play an important role in prolonging valve durability.

Here, we show that computational modeling based on preprocedural CT imaging may help mitigate potential downstream complications associated with THV underexpansion by facilitating selection of appropriate THV size and type and guiding appropriate balloon size if ad hoc BAV is necessary. Indeed, our computational modeling predicted regions prone to underexpansion, particularly the mid region of balloon-expandable THVs. Following BAV, significant improvements in THV expansion were observed across the different levels and were aligned with modeling predictions, including cases in which simulations predicted a minimal amount of benefit from postimplant BAV. Indeed, the ability to identify situations where postimplant BAV may be ineffective—as this additional procedural step comes with risks including annular rupture, leaflet tears, and conduction disturbances—appears critical. Identifying high-risk anatomies, such as highly calcified or elliptical annuli, will be important to prevent incomplete expansion or annular rupture that may result from inappropriate BAV or scenarios where postimplant BAV benefit is minimal. This prediction modeling may thus further highlight situations where THV postdilatation will not provide clinically meaningful benefit. For instance, if PVL or elevated gradients are the consequence of significant calcification or misalignment beyond underexpansion, other strategies such as PVL closure may be more appropriate than postimplant BAV. Furthermore, modeling could guide THV sizing in challenging anatomies such as bicuspid valves, where downsizing has been shown to be a promising approach with the potential to reduce the risk of THV underexpansion and limit aortic root mechanical stretch.^20^ Although the present study only had 1 patient with a bicuspid valve, previous studies on computational modeling of bicuspid valves have shown similar predictive performance.^21^

Interestingly, in our ViV cases, computational modeling also predicted substantial improvements following postimplant BAV, notably in constrained mid regions. In addition, computational modeling closely mirrored CT findings across valve types, with minor discrepancies noted primarily at the outflow. This supports the robustness of computational modeling in assessing procedural results and optimizing prosthesis sizing, particularly in complex ViV scenarios in which intraprocedurally assessing underexpansion can be challenging.

In addition, computational models predicting the effect of post-implant BAV may also be useful in redo-TAVI procedures. Indeed, assessment of the failed THV expansion is often a key issue and selecting the appropriate redo-THV size may be challenging.^22^ Thus, predicting expansion of the failed THV following postimplant BAV and before the redo-THV implant may lead to appropriate redo-THV sizing and thereby avoiding redo-THV underexpansion, which may be more challenging to address. Furthermore, simulating THV deployment at varying depths relative to the implantation level observed in post-CT to explore its effect on THV expansion and model predictive accuracy should be explored in future research.

Incorporating computational modeling into the procedural planning process may thus enhance the precision of THV selection and balloon sizing during the index procedure to prevent complications and improve immediate and long-term outcomes. Studies with larger sample sizes and long-term follow-ups will be needed to provide further insights into the impact of computational modeling on clinical decision-making and clinical outcomes. Future iterations of computational modeling should also include an addition of predilatation for valves or procedures that are anticipated to undergo this step. Future studies should incorporate dynamic hemodynamic modeling and structural analyses to better understand the functional implications of improved THV expansion. This combined approach may further enhance clinical decision-making and procedural planning accuracy and ultimately improve patient outcomes.

## Limitations

This study’s limitations mostly stem from its relatively limited sample size and single-center observational design, particularly in terms of the number of self-expandable devices studied. Because multiple outcomes were tested without formal adjustment for multiple comparisons, the risk of type I error is increased, and these findings should be interpreted as exploratory. This underscores the need for larger multicenter studies with extended follow-up periods. Moreover, post-TAVI and post-BAV hemodynamic performances were not incorporated into computational simulations, thus potentially missing dynamic interactions and flow mechanics. Notably, in the present cases, a noncompliant, high-pressure balloon was routinely favored to ensure homogenous diameters at all levels to facilitate symmetrical THV expansion from the inflow to the outflow. Furthermore, future iterations of the computational model should integrate the ability to predict the impact of delivery system underfilling as well as bioprosthetic valve fracture, as these variables are currently not accounted for. The ability of the computational model to predict effectiveness of postimplant BAV using a semicompliant balloon remains unknown as these may eventually adopt a less predictable “dog bone” shape, depending on areas of most resistance. Finally, the sample of patients arguably has a significant selection bias, as it consists of patients who underwent postimplant BAV for THV dysfunction is not compared to CTs of patients without THV underexpansion. This limitation reflects institutional practice, as routine CT follow-ups are not conducted; only patients that become symptomatic or have worsening echocardiographic parameters on routine transthoracic echocardiograms following their TAVI procedure undergo dedicated CT THV imaging. However, the corresponding impact appears mitigated by the fact that the computational modeling was performed in a blinded fashion while the model predicted (1) whether there would be underexpansion, (2) its degree of severity, and (3) the degree of efficacy/inefficacy of postimplant BAV.

## Conclusions

Predictive computational modeling has the potential to help select patients that could benefit from postimplant BAV to optimize THV expansion during the index TAVI procedure. Further studies are required to determine whether this approach may improve long-term THV function, reduce the need for late interventions, and mitigate the risks associated with unnecessary postimplant BAV in cases where it is expected to have no/minimal effect.

## Ethics Statement

This research has adhered to the relevant ethical guidelines.

## Funding

The authors have no funding to report.

## Disclosure Statement

Mariama Akodad is a consultant to 10.13039/100006520Edwards Lifesciences, 10.13039/100000046Abbott, and 10.13039/100004374Medtronic. Philipp Blanke is a consultant to 10.13039/100006520Edwards Lifesciences and provides CT core lab services for 10.13039/100006520Edwards Lifesciences, 10.13039/100004374Medtronic, Neovasc, Guided Delivery Systems, and 10.13039/100000046Abbott, for which no direct compensation is received. David Meier has received an institutional grant from 10.13039/100006520Edwards Lifesciences. Jonathon A. Leipsic holds institutional research core lab agreements with 10.13039/100004374Medtronic, 10.13039/100006520Edwards Lifesciences, 10.13039/100000046Abbott, 10.13039/100008497Boston Scientific, and Pi CARDIA. Stephanie L. Sellers is supported by a Michael Smith Health Research Scholar Award; is a consultant to 10.13039/100006520Edwards Lifesciences, 10.13039/100004374Medtronic, 10.13039/100000046Abbott, Anteris Technologies, and Excision Medical; has received research support from Edwards Lifesciences, Medtronic, and Anteris Technologies; and has stock options in Excision Medical. David A. Wood is a consultant to and has received research funding from 10.13039/100006520Edwards Lifesciences and 10.13039/100000046Abbott. John G. Webb is a consultant for 10.13039/100006520Edwards Lifesciences.

The other authors had no conflicts to declare.

## Supplementary Material

Supplementary docx 1
