# Supplementary material for: Prediction of Underexpansion Following Transcatheter Aortic Valve Implantation and Efficacy of Balloon Postdilatation
Source: Struct Heart. 2026 Apr 27;10(8):100850. doi: 10.1016/j.shj.2026.100850 (PMC13476896; doi:10.1016/j.shj.2026.100850)
Supplement: Supplementary file 1 [file mmc1.docx]

**Supplemental Material**

**Supplemental Methods**……………………………………………...……………..…………pg. 2

**Supplemental Table 1: Post-BAV procedure characteristics of the total population**……pg.6

**Supplemental Table 2: Patient characteristics, annular calcification, oversizing strategies for native TAVI**………………………………………………………………………….……pg.7

**Supplemental Table 3: Calcium volume measured from patient-specific anatomical reconstruction in native TAVI**………………………………………………………………pg.8

**Supplemental Figure 1: Computed tomography and predicted THV expansion segregated by native and valve-in-valve TAVI.**…...……………………………..…………….…..…….pg. 9

**Supplemental Figure 2: Accuracy of Simulated Expansion in Balloon-Expandable Transcatheter Heart Valves** …...…………………………….……………………..…….pg. 10

**Supplemental Figure 3: Mean gradient in relation to predicted and actual expansion of THVs following late balloon valvuloplasty.….......…………………………..….…..…….**pg. 11

**Supplemental Figure 4: Impact of Calcific Burden on Predictive Model Accuracy in Native TAVI** …...…………………………….…………………………………………….………..pg. 12

**Supplemental Methods**

**Predictive Modeling**

*Segmentation*

To simulate the deployment of the transcatheter heart valve, patient specific anatomical models of the aortic root, native or bioprosthetic valve leaflets, bioprosthetic stent and calcification were reconstructed from pre-TAVI MSCT imaging using segmentation methods (Figure 1) in Materialise Innovation Suite (Materialise, Leuven, Belgium). The aortic root was segmented by applying a Houndsfield Unit (HU) threshold of 226 to generate a mask and separating out the blood volume of only the ascending aorta, aortic valve and left ventricle using a multiple slice edit function. Post-processing of the aortic root was done by hollowing the aortic root lumen volume and trimming off the bottom of the ventricle and the top end of the ascending aorta. A reslice plane was defined perpendicular to the aortic annulus to better visualize the leaflet and/or bioprosthetic valve geometry. Native and bioprosthetic aortic valve leaflets were then segmented by removing the left ventricle from the aortic root mask and capturing only the lumen volume above the leaflets. A boolean subtraction method was applied to the leaflet mask to isolate the 3 native or bioprosthetic valve leaflets. Patients with thickened native/bioprosthetic leaflets were segmented directly from the CT images. Calcium nodules were reconstructed for the computational model from contrast-enhanced CT images by applying a variable thresholding window based on level of contrast, with an average minimum threshold of 582 ± 119 HU [406 – 791] and a maximum of 1506 ± 315 HU [739 - 2063]. Region grow method was used to isolate the nodules within the aortic root. Calcium volume was recorded after trimming any calcium volume above the native commissures, over 5 mm below the native annulus, and any calcium in the coronary arteries.

*Computational Materials and Simulation Setup*

The native aortic root, bioprosthetic aortic valve stent, native or bioprosthetic valve leaflets and calcification were meshed with tetrahedral volume elements with a target surface element length of 1.0 mm, 0.7mm, 0.5mm and 0.5mm respectively. The aortic root and bioprosthetic leaflets were prescribed a hyperelastic Ogden 3rd order model with coefficients shown in Table 1, fitted from benchtop results in literature [1, 2]. The bioprosthetic valve stent was modeled as linear elastic with ρ = 1400 kg/m^3, E = 1.9 GPa, ν = 0.4 [3]. The calcium nodules were prescribed elastic material properties (ρ = 1200 kg/m^3, E = 12.6 MPa, ν = 0.3) [4].

Table **1**. Soft tissue hyperelastic Ogden 3rd order model coefficients for aortic root and bioprosthetic leaflets.

| Part | α_1_ | α_2_ | α_3_ | µ_1_ | µ_2_ | µ_3_ | D_1_ | D_2_ | D_3_ |
| --- | --- | --- | --- | --- | --- | --- | --- | --- | --- |
| Root | -24.18 | -23.78 | -25.00 | -10.10 | 7.03 | 3.11 | 0.00 | 0.00 | 0.00 |
| Leaflets | 8.65 | 10.03 | 6.82 | -56.13 | 42.88 | 13.59 | 0.00 | 0.00 | 0.00 |
| Bioprosthetic Leaflets | 11.46 | 14.08 | 7.35 | -54.6 | 34.94 | 19.69 | 0.00 | 0.00 | 0.00 |

The SAPIEN stent was modeled using an elasto-plastic material model with a Young’s modulus of E = 193 GPa, σ_yield_ (0.2%) = 340 MPa, σ_u_ (48%) = 670 MPa and a Poisson’s ratio, ν = 0.29 with a density of ρ = 7760 kg/m^3^ [4]. The balloon for the deployment of the SAPIEN THV was modeled with a thickness of 0.06mm using membrane elements as a linear elastic material with E= 600 MPa, ν =0.45 and ρ = 1100 kg/m^3^ [4]. The Evolut stent was modeled using the inbuilt superelastic nitinol material model in Abaqus Explicit 2021. The parameters of the model were derived from in vitro testing of the Evolut device, as described in Finotello et. al. [5, 6]. The stent was assigned a density of 6450 kg/m^3^ and superelastic material properties of E_A_ = 44000 MPa, E_M_ = 20000 MPA, ν_A_ = 0.3, ν_M_ = 0.3, ε_L_ = 0.055, σ_L_^S^ = 460 MPa, σ_L_^E^ = 465 MPa, T_0_ = 37^o^C, σ_U_^S^ = 360 MPa, σ_U_^E^ = 260 MPa, σ_CL_^S^ = 900 MPa, ε_V_^L^ = 0.055.

All simulations of THV deployment were done targeting an 80-20 deployment for balloon-expanded THVs and 3mm below the annulus for self-expanding THVs. Information on the procedure such as implantation depth or occurrence of pre-dilatation was blinded from the simulation step to evaluate the predictive accuracy of the model as a pre-planning tool where information of procedure would not be available.

*Impact of Calcium Load on Simulation Accuracy*

To understand the potential impact of calcific burden on the predictive model’s accuracy, calcium volume was measured from the patient specific segmentations (Supplemental Table 3). Supplemental Figure 3 shows plots of the predictive accuracy of the model, represented by difference in predicted and CT-measured THV expansion post-TAVI at the inflow, mid and outflow regions, for balloon-expandable cases in native TAVR (n=9). The model showed good predictive accuracy regardless of calcium load, with no statistically significant correlation (p>0.05) observed between the predicted expansion accuracy and calcium volume at all 3 regions.

REFERENCES

1.	Pham T, Sulejmani F, Shin E, Wang D, Sun W. Quantification and comparison of the mechanical properties of four human cardiac valves. *Acta Biomater*, 2017, **54**:345-355.

2.	Yeats BB, Sivakumar SR, Samaee M, Polsani V, Yadav PK, Thourani VH, Sellers SL, Sathananthan J, Dasi LP. Calcium Fracture and Device Over Expansion in Transcatheter Aortic Valve Replacement for Bicuspid Aortic Valves*.* *Ann Biomed Eng*, 2023,**51:**2172-2181.

3.	Mayo RP, Yaakobovich H, Finkelstein A, Shadden SC, Marom G. Impact of BASILICA on the thrombogenicity potential of valve-in-valve implantations*.* *J Biomech*, 2021,**118**:110309.

4.	Tzamtzis S, Viquerat J, Yap J, Mullen MJ, Burriesci G. Numerical analysis of the radial force produced by the Medtronic-CoreValve and Edwards-SAPIEN after transcatheter aortic valve implantation (TAVI*).* *Med Eng Phys*. 2013,**35**:125-30.

5.	Finotello A, Gorla R, Brambila N, Bedogni F, Auricchio F, Morganti S. Finite element analysis of transcatheter aortic valve implantation: Insights on the modelling of self-expandable devices*.* *J Mech Behav Biomed Mater*, 2021,**123**:104772.

6.	Auricchio F, Taylor RL. Shape-memory alloys: Modelling and numerical simulations of the finite-strain superelastic behavior. *Computer Methods in Applied Mechanics and Engineering*, 1997. **143**:175-194.

**Supplemental Table 1: Post-BAV procedure characteristics of the total population.**

|  | **Total population**  **n = 20** |
| --- | --- |
| **Time THV implantation-post-dilatation** |  |
| - <1 month | 2 (10%) |
| - 1 month-1 year | 18 (90%) |
| - >1 year | 0 (0%) |
| Indication |  |
| - Elevated mean gradient | 12 (60%) |
| - Paravalvular leak | 7 (35%) |
| - Elevated mean gradient + PVL | 1 (5%) |
| Transfemoral access | 20 (100%) |
| Number of inflations |  |
| - 1 | 12 (60%) |
| - 2 | 4 (20%) |
| - >2 | 4 (20%) |

Values are n (%).

^a^PVL: paravalvular leak, ^b^THV: transcatheter aortic valve.

**Supplemental Table 2: Patient characteristics (n = 20), and annular calcification and oversizing strategies for native TAVI (n = 9).**

| **Females** | 6 (30%) |
| --- | --- |
| **Bicuspid aortic valve** | 1 (5%) |
| **Hypoattenuated leaflet thickening on pre-BAV CT** | 5 (25%) |
| **Comorbidities**  Hypertension  Dyslipidemia  Diabetes mellitus  Chronic obstructive pulmonary disease  Chronic kidney disease (eGFR < 60 mL/min)  Previous stroke  Previous pacemaker insertion  Permanent atrial fibrillation | 12 (60%)  12 (60%)  5 (25%)  6 (30%)  4 (20%)  2 (10%)  4 (20%)  3 (15%) |
| **Over-sizing % for native TAVI (n = 9)**  0-5%  5-10%  >10% | 4 (44%)  2 (22%)  3 (33%) |
| **Annular calcification for native TAVI (n = 9)**  None  Mild  Moderate  Severe | 4 (44%)  3 (33%)  2 (22%)  0 (0%) |

**Supplemental Table 3. Calcium volume measured from patient specific anatomical reconstruction in native TAVI.**

| **Case Number** | **THV device** | **Size** | **Calcium Volume (mm^3^)** |
| --- | --- | --- | --- |
| 6 | SAPIEN 3 | 26 mm | 904.70 |
| 7 | SAPIEN 3 | 23 mm | 215.37 |
| 8 | SAPIEN 3 | 26 mm | 617.97 |
| 9 | S3 Ultra | 26 mm | 736.76 |
| 14 | S3 Ultra | 23 mm | 619.58 |
| 15 | S3 Ultra | 26 mm | 423.04 |
| 17 | SAPIEN 3 | 29 mm | 1317.34 |
| 18 | S3 Ultra | 26 mm | 728.36 |
| 19 | S3 Ultra | 23 mm | 106.46 |

**Supplemental Figure 1:** Computed tomography and predicted THV expansion segregated by native and valve-in-valve TAVI.

**Supplemental Figure 2:** Accuracy of Simulated Expansion in Balloon-Expandable THVs

**Supplemental Figure 3.** Mean gradient in relation to predicted and actual expansion of THVs following late balloon valvuloplasty.

**Supplemental Figure 4.** Impact of Calcific Burden on Predictive Model Accuracy in Native TAVI.
